# Depth-Resolved Profile of the Interfacial Ferromagnetism in CaMnO_3_/CaRuO_3_ Superlattices

**DOI:** 10.1021/acs.nanolett.4c02087

**Published:** 2024-11-20

**Authors:** Jay R. Paudel, Aria Mansouri Tehrani, Michael Terilli, Mikhail Kareev, Joseph Grassi, Raj K. Sah, Liang Wu, Vladimir N. Strocov, Christoph Klewe, Padraic Shafer, Jak Chakhalian, Nicola A. Spaldin, Alexander X. Gray

**Affiliations:** † Physics Department, Temple University, Philadelphia, Pennsylvania 19122, United States; ‡ Materials Theory, ETH Zurich, Wolfgang-Pauli-Strasse 27, CH-8093 Zürich, Switzerland; § Department of Physics and Astronomy, Rutgers University, Piscataway, New Jersey 08854, United States; ∥ Swiss Light Source, Paul Scherrer Institute, 5232 Villigen, Switzerland; ⊥ Advanced Light Source, Lawrence Berkeley National Laboratory, Berkeley, California 94720, United States

**Keywords:** strongly correlated oxides, interfacial magnetism, X-ray spectroscopy, density functional theory

## Abstract

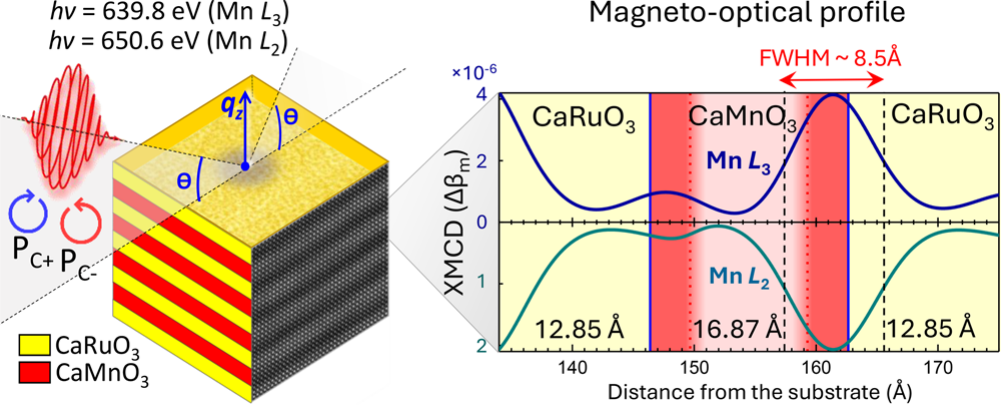

Emergent magnetic phenomena at interfaces represent a frontier in materials science, pivotal for advancing technologies in spintronics and magnetic storage. In this Letter, we utilize a suite of advanced X-ray spectroscopic and scattering techniques to investigate emergent interfacial ferromagnetism in oxide superlattices composed of antiferromagnetic CaMnO_3_ and paramagnetic CaRuO_3_. Our findings demonstrate that ferromagnetism exhibits an asymmetric profile and may extend beyond the interfacial layer into multiple unit cells of CaMnO_3_. Complementary density functional calculations reveal that the interfacial ferromagnetism is driven by the double exchange mechanism, facilitated by charge transfer from Ru to Mn ions. Additionally, defect chemistry, particularly the presence of oxygen vacancies, can play a crucial role in modifying the magnetic moments at the interface, possibly leading to the observed asymmetry between the top and bottom CaMnO_3_ interfacial magnetic layers. Our findings underscore the potential of manipulating interfacial ferromagnetism through point defect engineering.

The control of magnetic properties in oxide superlattices has attracted significant research interest due to their potential applications in spintronics.^1−5^ Specifically, the stabilization and control of interfacial ferromagnetic ground states in material systems composed of two nonferromagnetic materials hold significant importance from both a fundamental and technological perspective.^6−9^

The earliest and perhaps the best-known examples of such a material systems are oxide superlattices composed of antiferromagnetic CaMnO_3_ and paramagnetic CaRuO_3_ layers, which have been extensively studied for their ferromagnetic properties.^9−16^ In a pioneering study, Takahashi et al.^9^ demonstrated a ferromagnetic transition at approximately 95 K, localized near the interface region. The magnetization and magnetoconductance of the superlattice remained constant and independent of the varying thickness, indicating the crucial role of the interface in the observed ferromagnetic-like behavior.

A subsequent theoretical study^10^ explained this experimental observation by finding an exponential leakage of metallic Ru 3d e_g_ electrons across the interface into the insulating CaMnO_3_. This charge transfer was shown to stabilize the ferromagnetic state at the interface through ferromagnetic Anderson–Hasegawa double exchange,^17,18^ which competed with the antiferromagnetic superexchange in bulk CaMnO_3_ to form a one-unit-cell-thick ferromagnetic interfacial CaMnO_3_ layer. The calculations also indicated minimal electron penetration beyond the interfacial layer, explaining the bulk antiferromagnetism in the remaining CaMnO_3_.

Subsequent experiments yielded conflicting results regarding the size of the ferromagnetic unit cell. One experimental investigation, using a combination of spectroscopic probes, demonstrated that the aforementioned ferromagnetic polarization extends 3–4 unit cells (u.c.) into CaMnO_3_, surpassing the one-unit-cell limit and suggesting the presence of magnetic polarons at the interface.^12^ However, another study, employing polarized neutron reflectivity, revealed that interfacial ferromagnetism is indeed confined to only one unit cell of CaMnO_3_ at each interface.^14^ Moreover, it has been suggested that the magnitudes of the interfacial Mn magnetic moments could be modulated by changing the symmetry of oxygen octahedra connectivity at the boundary, thus proposing the tuning of interfacial symmetry as a new route to control emergent interfacial ferromagnetism.^16^

In this article, we present an in-depth analysis of interfacial ferromagnetism in CaMnO_3_/CaRuO_3_ superlattices, leveraging advanced synchrotron-based resonant X-ray reflectivity (XRR) techniques and density functional calculations to explore the magnetic properties at the interface of the two materials. We derive the detailed magneto-optical profile of the interfacial ferromagnetic layer and demonstrate that although it is centered in the interfacial unit cell of CaMnO_3_, it exhibits significant Gaussian-like broadening with a full width at half-maximum (FWHM) of approximately 8.5 Å, possibly extending beyond a single unit cell. Density functional calculations confirm that interfacial ferromagnetism is driven by a double exchange mechanism, facilitated by charge transfer from Ru to Mn across the interface, and show that oxygen vacancies alter Mn magnetic moments. Detailed fitting of the *q*_*z*_-dependent X-ray magnetic circular dichroism (XMCD) asymmetry spectra reveals pronounced magnetic asymmetry between the top and bottom magnetic interfaces. Our findings suggest that the presence of point defects, particularly oxygen vacancies, significantly influences the magnitude of the magnetic moments, offering a potential method to manipulate interfacial ferromagnetism in oxide superlattices for advanced spintronic applications.

A high-quality epitaxial superlattice consisting nominally of [4 u.c. CaMnO_3_/4 u.c. CaRuO_3_] × 10 was synthesized on a single-crystalline LaAlO_3_ (001) substrate using pulsed laser interval deposition.^19^ In-situ monitoring of layer-by-layer growth was conducted by using reflection high-energy electron diffraction (RHEED). The coherent epitaxy, crystallinity, and layering of the superlattice were verified through ex situ X-ray diffraction spectroscopy (XRD) and X-ray reflectivity (XRR). To confirm the correct elemental layering of the superlattice, standing-wave photoemission spectroscopy (SW-XPS)^20^ measurements were carried out at the soft-X-ray ARPES endstation^21^ of the high-resolution ADRESS beamline at the Swiss Light Source.^22^ The correct chemical composition was confirmed using bulk-sensitive hard X-ray photoelectron spectroscopy (HAXPES) measurements^23^ with a laboratory-based spectrometer. Furthermore, synchrotron-based soft X-ray resonant and nonresonant reflectivity measurements, described in detail later in this article (Figures 1 and 2), were used to determine the individual layer thicknesses and assess the interface quality. The characterization results of XRD, XRR, SW-XPS, and HAXPES are presented in Figures S1, S2, S3 and S4 of the Supporting Information (see also refs (24−29)).

**Figure 1:**
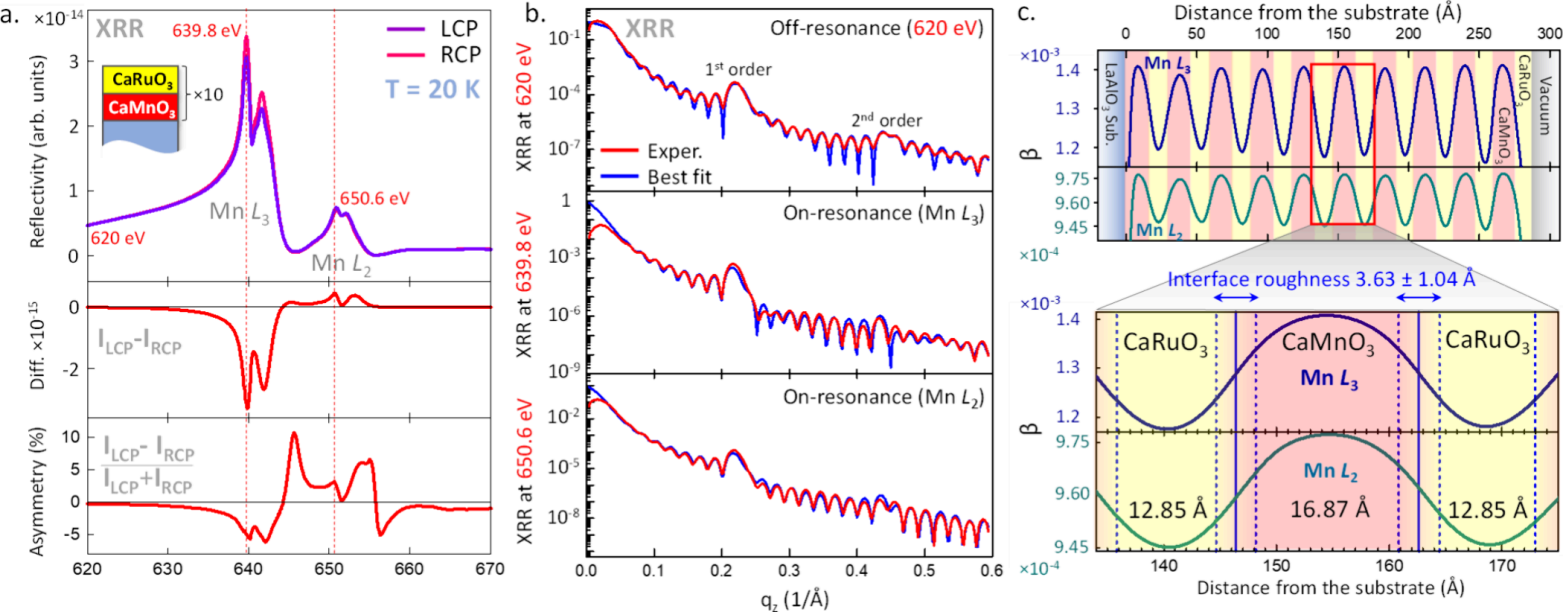
(a) Upper panel: circular polarization-dependent XRR energy scans across the Mn L_3_ and L_2_ absorption thresholds. The measurements were carried out at a constant value of momentum transfer *q*_*z*_ and at a temperature of 20 K. XRR XMCD difference (*I*_LCP_ – *I*_RCP_) and magnetic asymmetry (*I*_LCP_ – *I*_RCP_)/(*I*_LCP_ + *I*_RCP_) are shown in the lower panels. Three key photon energies corresponding to the nonresonant excitation (620 eV), the Mn L_3_ peak (639.8 eV), and the Mn L_2_ peak (650.6 eV) are marked with red dashed lines. (b) Momentum-dependent XRR spectra and the best fits to the experimental data measured at the three photon energies. Self-consistent fitting of the data yields a detailed optical absorption coefficient β profile of the sample, shown in (c), with the extracted layer thicknesses of 12.85 Å (CaRuO_3_) and 16.87 Å (CaMnO_3_), as well as the average interface roughness (chemical interdiffusion) of 3.63 ± 1.04 Å.

To derive the detailed X-ray optical depth profile as well as the element-specific (Mn) magneto-optical profile of the superlattice, we utilized polarization-dependent soft X-ray resonant and nonresonant reflectivity at the high-resolution (Δ*E* ≈ 100 meV) Magnetic Spectroscopy beamline 4.0.2 at the Advanced Light Source.^30^ All measurements were carried out in an applied in-plane magnetic field of 0.1 T and at the sample temperature of 20 K, which is well below the reported *T*_c_ (∼95 K) for this system.^9^

Figure 1a shows circular polarization-dependent XRR energy scans across the Mn L_3_ and L_2_ absorption edge carried out at a constant value of momentum transfer *q*_*z*_ in specular X-ray incidence geometry. The XMCD difference (*I*_LCP_ – *I*_RCP_) and percent magnetic asymmetry (*I*_LCP_ – *I*_RCP_/*I*_LCP_ + *I*_RCP_) are shown in the bottom panels and indicate ferromagnetism on the Mn sites. These data, measured in specular reflectivity mode, can be compared to the standard X-ray absorption (XAS) and XMCD spectra recorded in the total electron yield (TEY) mode of acquisition on the same sample, as shown in Figure S5a of the Supporting Information. These spectra show excellent agreement with the prior XAS studies of the CaMnO_3_/CaRuO_3_ superlattices.^12,16^ Furthermore, as in these prior studies, they reveal fine spectral features attributed to Mn^3+^ and Mn^4+^ cations. This suggests a mixed Mn valence state in CaMnO_3_, which is required for the Mn^3+^–Mn^4+^ ferromagnetic double exchange interaction.^18^ An additional reference XAS measurement of a bulk-like 30 nm thick CaMnO_3_ film grown on an LaAlO_3_ substrate was carried out using the bulk-sensitive luminescence yield (LY) detection mode. The spectrum, shown in Figure S6 of the Supporting Information, exhibits a line shape characteristic of a predominantly Mn^4+^ valence state, with only a minor Mn^3+^-like component on the lower-photon-energy side. This suggests that the reduced (3+) Mn state observed in the superlattice samples is likely due to interfacial effects rather than intrinsic oxygen deficiency from the growth process. Similar bulk-sensitive XAS LY measurements were also carried out on the superlattice samples (Figure S7b).

To derive the detailed X-ray optical depth profile of the superlattice, we selected three photon energies corresponding to the off-resonant (620 eV) and resonant (Mn L_3_ at 639.8 eV and L_2_ at 650.6 eV) conditions and carried out *q*_*z*_-dependent specular XRR scans that are shown in Figure 1b (red curves). The photon energies mentioned above were selected by identifying the strongest peaks in the fixed-*q*_*z*_ X-ray reflectivity and XMCD spectra (Figure 1a). Notably, the *q*_*z*_-dependent specular XRR spectra shown in Figure 1b span a wide range of *q*_*z*_ (0–0.6 1/Å), encompassing both the first-order and second-order Bragg conditions (at ∼0.22 and ∼0.43 1/Å, respectively) and, therefore, contain detailed depth-resolved information on both the layering and the interfacial structure of the sample.^31^

The *q*_*z*_-dependent specular XRR spectra shown in Figure 1b (red curves) were fitted self-consistently with the XRR analysis program ReMagX,^32^ using an algorithm based on the Parratt formalism^33^ and the Névot–Croce interdiffusion approximation.^34^ For off-resonant spectrum fitting, only the thicknesses of the CaMnO_3_ and CaRuO_3_ layers and the interdiffusion lengths between them were allowed to vary. The resonant X-ray optical constants needed for calculations were obtained by a Kramers–Kronig analysis of the XAS data. These values served as starting input parameters for the resonant XRR analysis and were optimized (consistently for the L_3_ and L_2_ edges) during fitting. The blue spectra in Figure 1b represent the best theoretical fits to the experimental data, demonstrating exceptional agreement in terms of the amplitudes of all features as well as their relative phases and shapes.

A self-consistent X-ray optical profile of the superlattice resulting from the fitting of the three *q*_*z*_-dependent specular XRR spectra is shown in Figure 1c. The profile is represented as the depth-dependent (*x*-axis) variation of the absorption coefficient β at the photon energies corresponding to the Mn L_3_ (blue curve) and Mn L_2_ (green curve) edges. The maxima in such element-selective (Mn) absorption profiles correspond to the depth-resolved positions of the CaMnO_3_ layers and the minima to the positions of the CaRuO_3_ layers, where Mn is absent.

The lower part of Figure 1c presents a magnified view of the typical X-ray optical profile centered around a CaMnO_3_ layer roughly midway through the superlattice. The individual layer thicknesses obtained from the X-ray optical fitting are 12.85 Å for CaRuO_3_ and 16.87 Å for CaMnO_3_. These values correspond to approximately 3.5 and 4.5 primitive cubic unit cells of CaRuO_3_ and CaMnO_3_, respectively, using the lattice constants from prior studies.^10,20,35^ The average interface roughness (interdiffusion) is 3.63 ± 1.04 Å, corresponding to approximately one primitive cubic unit cell of a typical perovskite oxide. The total superlattice period of 29.72 Å corresponds precisely to 8 primitive cubic unit cells, matching lab-based XRR and synchrotron-based SW-XPS characterization shown in Supporting Figures S1 and S2. Minor deviations in calculated layer thicknesses may arise from slight inaccuracies in the resonant X-ray optical properties used as X-ray optical constants vary drastically near the Mn L_2,3_ resonances.

Thus, we have demonstrated that polarization-averaged *q*_*z*_-dependent specular XRR measurements combined with X-ray optical modeling enables determination of the X-ray optical profile of our CaMnO_3_/CaRuO_3_ superlattice, which also corresponds to the chemical/structural profile due to the use of element-specific (Mn) resonant photon energies. Building on this, we used the extracted chemical/structural profile as input in the model for fitting the *q*_*z*_-dependent XMCD asymmetry (*I*_LCP_ – *I*_RCP_/*I*_LCP_ + *I*_RCP_) spectra shown in Figure 2a. Since these magnetic asymmetry spectra are derived from the same reflectivity data used for the chemical/structural analysis, this method self-consistently constrains the model, allowing sensitive determination of the depth-resolved magneto-optical profile.

**Figure 2:**
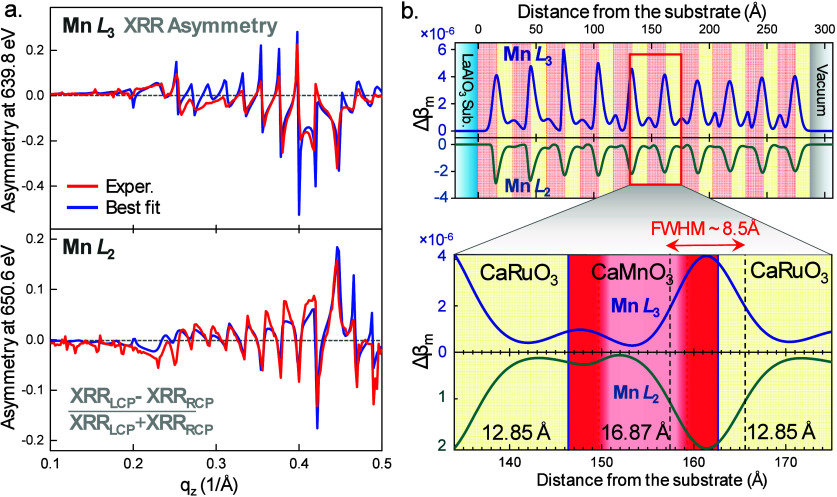
(a) *q*_*z*_-dependent XMCD asymmetry spectra and the best fits to the experimental data measured at the resonant photon energies of the Mn L_3_ (639.8 eV) and Mn L_2_ (650.6 eV) XRR peaks. Self-consistent fitting of the data yields the detailed magneto-optical profile of the sample shown in (b). (b) Depth-resolved magneto-optical profile given by the modulation of the magnetic dichroism of the X-ray absorption coefficient Δβ_m_. The expanded region in the bottom panel reveals an asymmetry in the magnetic moment at the top and bottom CaMnO_3_ interfaces. The interfacial ferromagnetic layer exhibits a characteristic Névot–Croce (Gaussian-like) profile with a FWHM of approximately 8.5 Å centered in the interfacial unit cells of CaMnO_3_.

We used data collected at the photon energies of both Mn L_3_ (top panel) and L_2_ (bottom panel) edges to further constrain the fitting. The only three variable parameters were the thickness and roughness of the interfacial magnetic layer and the X-ray optical constant Δβ_m_, which quantifies the magnitude of the modulation of the magnetic dichroism of the X-ray absorption coefficient β. Notably, the use of *q*_*z*_-dependent XMCD asymmetry spectra significantly enhances the sensitivity of the fitting due to the intricate spectral line shapes, as depicted in Figure 2a, and the numerous sharp modulations with varying amplitudes and shapes across the entire *q*_*z*_ range. This improvement is in contrast to the traditional use of unnormalized *q*_*z*_-dependent XMCD difference (*I*_LCP_ – *I*_RCP_) spectra, as is commonly seen in similar studies.

The resultant magneto-optical profiles, characterized by the thickness-dependent modulations of the values of Δβ_m_ at the resonant energies of Mn L_3_ (positive values, shown in blue) and Mn L_2_ (negative values shown in green), are depicted in Figure 2b. The opposite signs are in agreement with the traditional convention for representing XMCD signals at the L_3_ and L_2_ edges. The difference in the amplitudes between the Mn L_3_-derived and Mn L_2_-derived profiles is also consistent with expected XMCD signal differences at these two absorption edges (see Figure S5 in the Supporting Information).

The expanded region of Figure 2b shows the detailed magneto-optical profile of the CaMnO_3_ layer, and the two adjacent CaRuO_3_ layers, in the superlattice’s central region. The most striking feature is the several-fold (×5.5) asymmetry between signals at the bottom (CaRuO_3_/CaMnO_3_) and top (CaMnO_3_/CaRuO_3_) interfaces, which will be discussed shortly. The maxima of the magnetic signal are centered almost perfectly in the interfacial unit cells of CaMnO_3_. However, the estimated thickness of the magnetic layer, calculated from the full width at half-maximum (FWHM) of the Δβ_m_ profile shown in Figure 2b, is approximately 8.5 Å, corresponding to approximately 2.3 primitive cubic unit cells of CaMnO_3_. Significant broadening of the magnetic signal, modeled by the Névot–Croce-type interdiffusion,^34^ appears on both sides of the magnetic layer. On the side of the CaMnO_3_ layer, this indicates a possible extension of the magnetic signal into adjacent CaMnO_3_ unit cells with gradually decreasing intensity, as there is no sharp transition from ferromagnetic to nonferromagnetic regions within the CaMnO_3_ layer. On the other side, where the CaMnO_3_ interfaces with the CaRuO_3_ layer, the observed broadening is also expected, mainly due to chemical interdiffusion of Mn or intermixing, common in such material systems and, in this case, was estimated to be about one unit cell wide (see Figure 1c).

Therefore, although the ferromagnetism is clearly strongest in the interfacial unit cell of CaMnO_3_, the total extent of the ferromagnetic signal is in the range of 1–2.3 cubic unit cells. This finding bridges discrepancies between studies that observe (or predict) interfacial ferromagnetism confined to a single interfacial unit cell of CaMnO_3_^10,14^ and those showing it extends several unit cells from the interface,^12^ as the definition of the magnetic layer thickness can significantly affect its quantification.

Since the maximum available applied magnetic field in XRR measurements (0.1 T) was below the reported saturation field for the CaMnO_3_/CaRuO_3_ ferromagnetic interface (∼1 T),^9^ it was necessary to confirm the observed difference (asymmetry) between the magnitudes of the magnetic signal at the top and bottom CaMnO_3_ interfaces at a higher applied field. Thus, we conducted a comparative study using XAS/XMCD in TEY detection mode with a 4 T field. We compared our original sample, terminated with the CaRuO_3_ layer, to a superlattice sample synthesized in the same batch but terminated with reversed layers, specifically with the CaMnO_3_ layer instead of CaRuO_3_.

The TEY is a more surface-sensitive modality of XAS, with an average probing depth of 2–5 nm, decaying exponentially from the surface into the bulk.^36,37^ Therefore, the XMCD measurement of the original CaMnO_3_/CaRuO_3_ sample (terminated with CaRuO_3_) is most sensitive to the CaMnO_3_/CaRuO_3_ (“top” type) interface. Conversely, the measurement of the CaRuO_3_/CaMnO_3_ sample (terminated with CaMnO_3_) is most sensitive to the CaRuO_3_/CaMnO_3_ (“bottom” type) interface. Measurements reveal a significantly weaker (×2.7) magnetic signal for the CaRuO_3_/CaMnO_3_ (“bottom” type) interface compared to the CaMnO_3_/CaRuO_3_ (“top” type) interface, qualitatively consistent with our reflectivity measurements (Figure S5 of the Supporting Information). We speculate that, due to the signal’s exponential decay with depth, there is still some contribution to the total magnetic signal from the lower (CaMnO_3_/CaRuO_3_) interface, resulting in weaker suppression of the depth-averaged magnetic signal (×2.7) compared to the depth-resolved XRR measurements (×5.5).

As additional *theoretical* verification of the observed asymmetry, we repeated the fitting of the *q*_*z*_-dependent XMCD asymmetry spectra using the same model, but with an additional constraint forcing Δβ_m_ magnitudes to be the same for both interfaces. This modification resulted in a drastic deterioration in the quality of the fit (Figure S8 in the Supporting Information).

To explore the origin of the ferromagnetism at the interface, we performed DFT calculations of the structure and electronic properties of CaMnO_3_/CaRuO_3_ superlattices (see Supporting Information).^38,39^ First, we calculated the energy for three magnetic configurations—the entire CaMnO_3_ slab set to its bulk G-type antiferromagnetic (AFM) structure, the interfacial CaMnO_3_ layers set to be ferromagnetic (FM) with the middle layers constrained to G-type AFM, and the entire CaMnO_3_ slab set to be FM, rerelaxing the structure in each case. We found that the lowest energy arrangement has the G-type AFM ordering of the bulk in the central region of CaMnO_3_, with FM favored at the interface, consistent with our measurements; the relative energies are shown in Figure 3b. Interestingly, interfacial FM is so strongly favored that it is lower in energy for the entire CaMnO_3_ slab to adopt the FM configuration than for it all to have the AFM configuration of the bulk.

**Figure 3:**
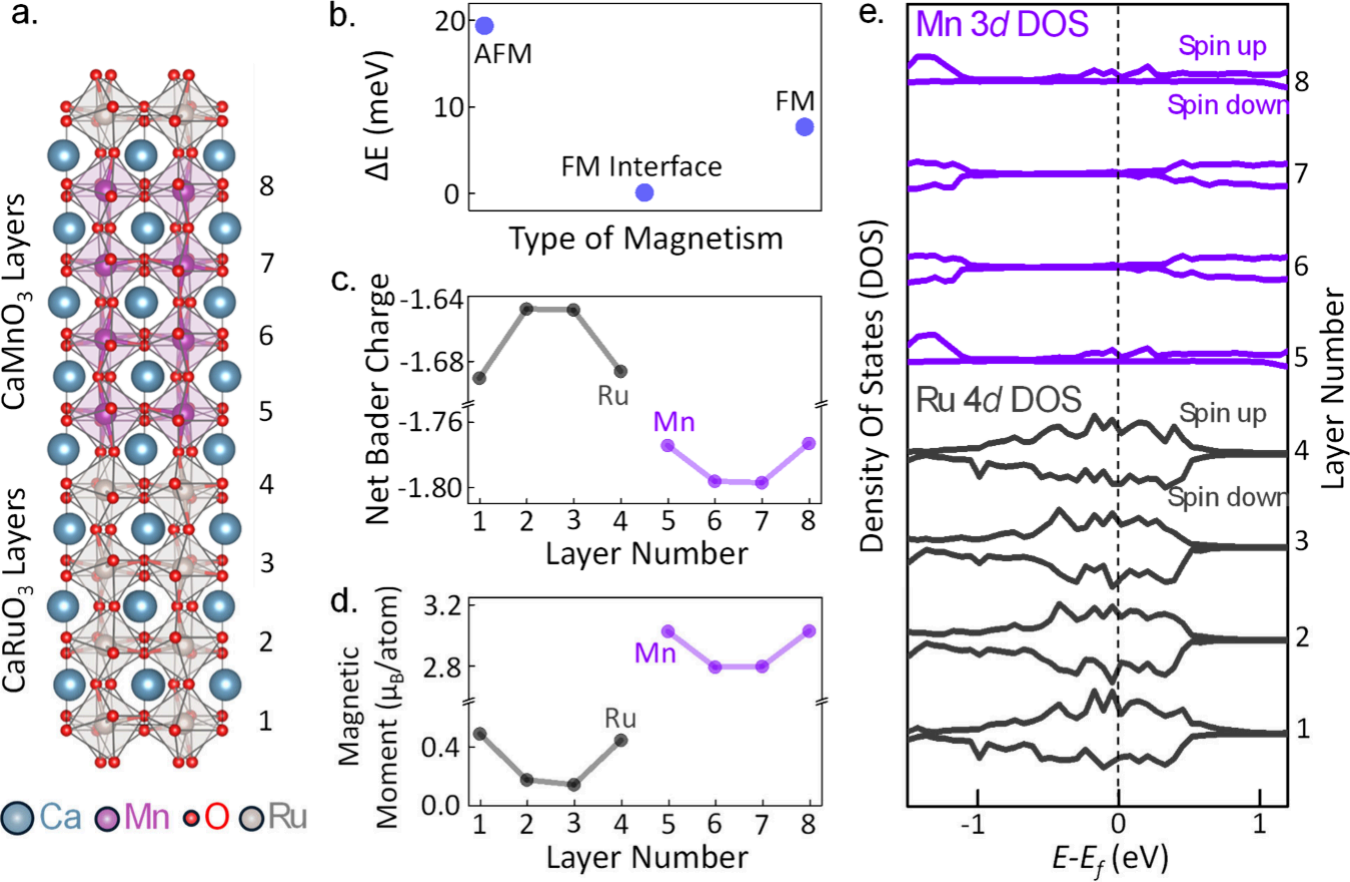
(a) Crystal structure of the CaRuO_3_/CaMnO_3_ supercell (Ca in blue, Mn in magenta, O in red, Ru in gray) after ionic relaxation. (b) Calculated energy differences between various magnetic states of the CaMnO_3_ layers within the supercell: AFM denotes the entire 4-unit cell slab in an antiferromagnetic state; FM Interface indicates that only one unit cell at the interface exhibits ferromagnetism while the remaining bulk retains a bulk-like antiferromagnetic state; FM represents the entire CaMnO_3_ slab in a ferromagnetic state. (c) Net Bader charges for the individual layers of CaRuO_3_ and CaMnO_3_ in the supercell. (d) Layer-resolved magnetic moments per atom for the Ru atoms in CaRuO_3_ and the Mn atoms in CaMnO_3_. (e) Partial spin-projected densities of states for the Ru 4d states in the CaRuO_3_ layers and for the Mn 3d states in the CaMnO_3_ layers.

Having established that our calculations reproduce our measured interfacial magnetism, we examined its origin. To this end, we calculated the layer resolved transition-metal Bader charges, magnetic moments, and densities of states; our results are shown as a function of layer number in Figure 3c–e, respectively, with Ru values in black and Mn values in blue. Our calculated Bader charges (Figure 3c) show charge transfer from Ru to Mn layers at the interface, consistent with the increased interfacial local Mn magnetic moment (Figure 3d) and the metallic partial density of states (Figure 3e). Therefore, our calculations point to a double exchange mechanism driven by interfacial metallicity as the origin of ferromagnetism, as proposed in refs (8) and (10).

We note that the top and bottom interfaces in our supercells are identical by symmetry, so our calculations using the nominal superlattice structure do not capture the measured asymmetry between the magnetism of the top and bottom CaMnO_3_ interfacial layers. To explore the possible role of defect chemistry in this asymmetry, we repeated our calculation procedure for a supercell containing oxygen vacancies at one interface. Specifically, we remove one of the four oxygen atoms between the Mn and Ru atoms at one interface, as shown in Figure 4a. The resulting calculated magnetic moment per transition metal ion is substantially increased in the interfacial CaMnO_3_ layer containing the vacancy (see Figure 4b), pointing to a difference in the point defect chemistry, which could be introduced during the growth process as the possible origin of the different sizes of the ferromagnetic moments at the two interfaces. Notably, other structural and electronic factors, not considered in this study, could also contribute to the observed magnetic asymmetry.

**Figure 4:**
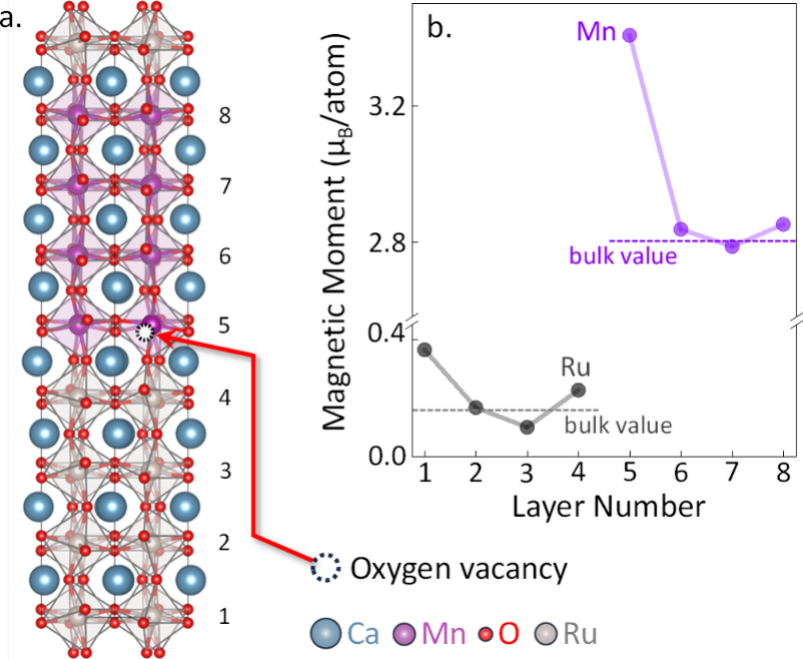
(a) Crystal structure of the CaRuO_3_/CaMnO_3_ supercell with an oxygen vacancy introduced for one of the O atoms intermediate between the Mn and Ru atoms. (b) Layer-resolved magnetic moments per atom exhibiting significant asymmetry between the top and bottom interfaces, with the increased magnetic moment in the interfacial CaMnO_3_ layer that contains the vacancy. Dashed horizontal lines indicate the bulk-like values of the magnetic moments.

To rule out some of the other possible origins of the observed magnetic asymmetry between the top and bottom CaMnO_3_ interfacial layers, we repeated calculations for supercells with several plausible deviations from the structure shown in Figure 3a. Specifically, we considered structures with mixed [001] and [110] oxygen octahedral tilt patterns leading to frustrated octahedral tilt connectivity at the interface and superlattices with odd numbers of primitive cubic unit cell layers of CaMnO_3_ and CaRuO_3_. In each case, our calculations showed no significant magnetic asymmetry between the top and bottom interfacial CaMnO_3_ layers (Figure S9 in the Supporting Information).

In summary, we have discovered that the emergent ferromagnetism in CaMnO_3_/CaRuO_3_ oxide superlattices presents an asymmetric distribution and may extend beyond the interfacial layer, suggesting a more complex interfacial behavior than previously recognized. Density functional calculations indicate that this ferromagnetism is driven by a double exchange mechanism, attributed to charge transfer from Ru to Mn ions, with defect chemistry—such as oxygen vacancies—possibly playing an important role in creating the magnetic asymmetry observed at the interfaces. By pushing the boundaries of traditional magnetic interface studies and providing deeper and more detailed insight into the atomic-level interactions at these interfaces, this work paves the way for future innovations in magnetic storage and spintronics.

## Supporting Information Available

Lab-based and synchrotron-based characterization measurements, XRD, XRR, HAXPES, XAS and XMCD spectra, schematic diagram of XPS experiment, best fits of rocking curves, crystal structure, *q*_*z*_-dependent XMCD asymmetry spectra modeling, and DFT calculations (PDF)

## References

[ref1] Zubko P.; Gariglio S.; Gabay M.; Ghosez P.; Triscone J.-M. Interface Physics in Complex Oxide Heterostructures. Annu. Rev. Condens. Matter Phys. 2011, 2, 141–165. 10.1146/annurev-conmatphys-062910-140445.

[ref2] Bhattacharya A.; May S. J. Magnetic Oxide Heterostructures. Annu. Rev. Mater. Res. 2014, 44, 65–90. 10.1146/annurev-matsci-070813-113447.

[ref3] Hellman F.; et al. Interface-induced phenomena in magnetism. Rev. Mod. Phys. 2017, 89, 025006 10.1103/RevModPhys.89.025006. 28890576 PMC5587142

[ref4] Chen H.; Millis A. J. Charge transfer driven emergent phenomena in oxide heterostructures. J. Phys.: Condens. Matter 2017, 29, 243001 10.1088/1361-648X/aa6efe. 28437253

[ref5] Ramesh R.; Schlom D. G. Creating emergent phenomena in oxide superlattices. Nat. Rev. Mater. 2019, 4, 257 10.1038/s41578-019-0095-2.

[ref6] Yamada H.; Ogawa Y.; Ishii Y.; Sato H.; Kawasaki M.; Akoh H.; Tokura Y. Engineered Interface of Magnetic Oxides. Science 2004, 305, 646 10.1126/science.1098867. 15286367

[ref7] Oja R.; Tyunina M.; Yao L.; Pinomaa T.; Kocourek T.; Dejneka A.; Stupakov O.; Jelinek M.; Trepakov V.; van Dijken S.; Nieminen R. M. ***d***^0^ Ferromagnetic Interface between Nonmagnetic Perovskites. Phys. Rev. Lett. 2012, 109, 127207 10.1103/PhysRevLett.109.127207. 23005984

[ref8] Grutter A. J.; Yang H.; Kirby B. J.; Fitzsimmons M. R.; Aguiar J. A.; Browning N. D.; Jenkins C. A.; Arenholz E.; Mehta V. V.; Alaan U. S.; Suzuki Y. Interfacial Ferromagnetism in LaNiO_3_/CaMnO_3_ Superlattices. Phys. Rev. Lett. 2013, 111, 087202 10.1103/PhysRevLett.111.087202. 24010469

[ref9] Takahashi K. S.; Kawasaki M.; Tokura Y. Interface ferromagnetism in oxide superlattices of CaMnO_3_/CaRuO_3_. Appl. Phys. Lett. 2001, 79, 1324 10.1063/1.1398331.

[ref10] Nanda B. R. K.; Satpathy S.; Springborg M. S. Electron Leakage and Double-Exchange Ferromagnetism at the Interface between a Metal and an Antiferromagnetic Insulator: CaRuO_3_/CaMnO_3_. Phys. Rev. Lett. 2007, 98, 216804 10.1103/PhysRevLett.98.216804. 17677800

[ref11] Yamada H.; Sato H.; Akoh H.; Kida N.; Arima T.; Kawasaki M.; Tokura Y. Optical magnetoelectric effect at CaRuO_3_-CaMnO_3_ interfaces as a polar ferromagnet. Appl. Phys. Lett. 2008, 92, 062508 10.1063/1.2857466.

[ref12] Freeland J. W.; Chakhalian J.; Boris A. V.; Tonnerre J.-M.; Kavich J. J.; Yordanov P.; Grenier S.; Zschack P.; Karapetrova E.; Popovich P.; Lee H. N.; Keimer B. Charge transport and magnetization profile at the interface between the correlated metal CaRuO_3_ and the antiferromagnetic insulator CaMnO_3_. Phys. Rev. B 2010, 81, 094414 10.1103/PhysRevB.81.094414.

[ref13] Yordanov P.; Boris A. V.; Freeland J. W.; Kavich J. J.; Chakhalian J.; Lee H. N.; Keimer B. Far-infrared and dc magnetotransport of CaMnO_3_-CaRuO_3_ superlattices. Phys. Rev. B 2011, 84, 045108 10.1103/PhysRevB.84.045108.

[ref14] He C.; Grutter A. J.; Gu M.; Browning N. D.; Takamura Y.; Kirby B. J.; Borchers J. A.; Kim J. W.; Fitzsimmons M. R.; Zhai X.; Mehta V. V.; Wong F. J.; Suzuki Y. Interfacial Ferromagnetism and Exchange Bias in CaRuO_3_/CaMnO_3_ Superlattices. Phys. Rev. Lett. 2012, 109, 197202 10.1103/PhysRevLett.109.197202. 23215420

[ref15] Grutter A. J.; Kirby B. J.; Gray M. T.; Flint C. L.; Alaan U. S.; Suzuki Y.; Borchers J. A. Electric Field Control of Interfacial Ferromagnetism in CaMnO_3_/CaRuO_3_ Heterostructures. Phys. Rev. Lett. 2015, 115, 047601 10.1103/PhysRevLett.115.047601. 26252708

[ref16] Grutter A. J.; Vailionis A.; Borchers J. A.; Kirby B. J.; Flint C. L.; He C.; Arenholz E.; Suzuki Y. Interfacial Symmetry Control of Emergent Ferromagnetism at the Nanoscale. Nano Lett. 2016, 16, 5647 10.1021/acs.nanolett.6b02255. 27472285

[ref17] Anderson P. W.; Hasegawa H. Considerations on Double Exchange. Phys. Rev. 1955, 100, 675 10.1103/PhysRev.100.675.

[ref18] Briático J.; Alascio B.; Allub R.; Butera A.; Caneiro A.; Causa M. T.; Tovar M. Double exchange interaction in CaMnO_3-δ_. Czech. J. Phys. 1996, 53, 14020 10.1103/PhysRevB.53.14020. 9983188

[ref19] Kareev M.; Prosandeev S.; Gray B.; Liu J.; Ryan P.; Kareev A.; Moon E. J.; Chakhalian J. Sub-monolayer nucleation and growth of complex oxides at high supersaturation and rapid flux modulation. J. Appl. Phys. 2011, 109, 114303 10.1063/1.3590146.

[ref20] Chandrasena R. U.; Flint C. L.; Yang W.; Arab A.; Nemšák S.; Gehlmann M.; Özdöl V. B.; Bisti F.; Wijesekara K. D.; Meyer-Ilse J.; Gullikson E.; Arenholz E.; Ciston J.; Schneider C. M.; Strocov V. N.; Suzuki Y.; Gray A. X. Depth-resolved charge reconstruction at the LaNiO_3_/CaMnO_3_ interface. Phys. Rev. B 2018, 98, 155103 10.1103/PhysRevB.98.155103.

[ref21] Strocov V. N.; Wang X.; Shi M.; Kobayashi M.; Krempasky J.; Hess C.; Schmitt T.; Patthey L. Soft-X-ray ARPES facility at the ADRESS beamline of the SLS: concepts, technical realization and scientific applications. J. Synchrotron Rad. 2014, 21, 32–44. 10.1107/S1600577513019085. 24365914

[ref22] Strocov V. N.; Schmitt T.; Flechsig U.; Schmidt T.; Imhof A.; Chen Q.; Raabe J.; Betemps R.; Zimoch D.; Krempasky J.; Wang X.; Grioni M.; Piazzalunga A.; Patthey L. High-resolution soft X-ray beamline ADRESS at the Swiss Light Source for resonant inelastic X-ray scattering and angle-resolved photoelectron spectroscopies. J. Synchrotron Rad. 2010, 17, 631–643. 10.1107/S0909049510019862. PMC2927903 20724785

[ref23] Gray A. X.; Papp C.; Ueda S.; Balke B.; Yamashita Y.; Plucinski L.; Minar J.; Braun J.; Ylvisaker E. R.; Schneider C. M.; Pickett W. E.; Ebert H.; Kobayashi K.; Fadley C. S. Probing bulk electronic structure with hard X-ray angle-resolved photoemission. Nat. Mater. 2011, 10, 759 10.1038/nmat3089. 21841798

[ref24] Flint C. L.; Jang H.; Lee J.-S.; N’Diaye A. T.; Shafer P.; Arenholz E.; Suzuki Y. Role of polar compensation in interfacial ferromagnetism of LaNiO_3_/CaMnO_3_ superlattices. Phys. Rev. Mater. 2017, 1, 024404 10.1103/PhysRevMaterials.1.024404.

[ref25] Kuo C.-T.; Conti G.; Rault J. E.; Schneider C. M.; Nemšàk S.; Gray A. X. Emergent phenomena at oxide interfaces studied with standing-wave photoelectron spectroscopy. J. Vac. Sci. Technol. A 2022, 40, 020801 10.1116/6.0001584.

[ref26] Yang S.-H.; Gray A. X.; Kaiser A. M.; Mun B. S.; Sell B. C.; Kortright J. B.; Fadley C. S. Making use of X-ray optical effects in photoelectron-, Auger electron-, and X-ray emission spectroscopies: Total reflection, standing-wave excitation, and resonant effects. J. Appl. Phys. 2013, 113, 073513 10.1063/1.4790171.

[ref27] Karslıoǧlu O.; Gehlmann M.; Müller J.; Nemšák S.; Sethian J. A.; Kaduwela A.; Bluhm H.; Fadley C. S. An Efficient Algorithm for Automatic Structure Optimization in X-ray Standing-Wave Experiments. J. Electron Spectrosc. Relat. Phenom. 2019, 230, 10–20. 10.1016/j.elspec.2018.10.006.

[ref28] Fadley C. S.; Shirley D. A. Multiplet Splitting of Metal-Atom Electron Binding Energies. Phys. Rev. A 1970, 2, 1109–1120. 10.1103/PhysRevA.2.1109.

[ref29] Galakhov V. R.; Demeter M.; Bartkowski S.; Neumann M.; Ovechkina N. A.; Kurmaev E. Z.; Lobachevskaya N. I.; Mukovskii Y. M.; Mitchell J.; Ederer D. L. Mn 3*s* exchange splitting in mixed-valence manganites. Phys. Rev. B 2002, 65, 113102 10.1103/PhysRevB.65.113102.

[ref30] Young A. T.; Arenholz E.; Feng J.; Padmore H.; Marks S.; Schlueter R.; Hoyer E.; Kelez N.; Steier C. A soft X-ray undulator beamline at the Advanced Light Source with circular and variable linear polarization for the spectroscopy and microscopy of magnetic materials. Surf. Rev. Lett. 2002, 09, 549–554. 10.1142/S0218625X02002622.

[ref31] Benckiser E.; Haverkort M. W.; Bruck S.; Goering E.; Macke S.; Frano A.; Yang X.; Andersen O. K.; Cristiani G.; Habermeier H.-U.; Boris A. V.; Zegkinoglou I.; Wochner P.; Kim H.-J.; Hinkov V.; Keimer B. Orbital reflectometry of oxide heterostructures. Nat. Mater. 2011, 10, 189–193. 10.1038/nmat2958. 21297622

[ref32] Macke S.; Goering E. Magnetic reflectometry of heterostructures. J. Phys.: Condens. Matter 2014, 26, 363201 10.1088/0953-8984/26/36/363201. 25121937

[ref33] Parratt L. G. Surface Studies of Solids by Total Reflection of X-Rays. Phys. Rev. 1954, 95, 359 10.1103/PhysRev.95.359.

[ref34] Névot L.; Croce P. Study of thin layers and surfaces by grazing, specular or diffuse reflection of X-rays. Rev. Phys. Appl. (Paris) 1976, 11, 113 10.1051/rphysap:01976001101011300.

[ref35] Paudel J. R.; Terilli M.; Wu T.-C.; Grassi J. D.; Derrico A. M.; Sah R. K.; Kareev M.; Wen F.; Klewe C.; Shafer P.; Gloskovskii A.; Schlueter C.; Strocov V. N.; Chakhalian J.; Gray A. X. Direct experimental evidence of tunable charge transfer at the LaNiO_3_/CaMnO_3_ ferromagnetic interface. Phys. Rev. B 2023, 108, 054441 10.1103/PhysRevB.108.054441.

[ref36] Nakajima R.; Stöhr J.; Idzerda Y. U. Electron-yield saturation effects in *L*-edge X-ray magnetic circular dichroism spectra of Fe, Co, and Ni. Phys. Rev. B 1999, 59, 6421 10.1103/PhysRevB.59.6421.

[ref37] Jablonski A.; Powell C. J. Practical expressions for the mean escape depth, the information depth, and the effective attenuation length in Auger-electron spectroscopy and X-ray photoelectron spectroscopy. J. Vac. Sci. Technol. A 2009, 27, 253 10.1116/1.3071947.

[ref38] Kresse G.; Furthmüller J. Efficient iterative schemes for *ab initio* total-energy calculations using a plane-wave basis set. Phys. Rev. B 1996, 54, 11169 10.1103/PhysRevB.54.11169. 9984901

[ref39] Dudarev S. L.; Botton G. A.; Savrasov S. Y.; Humphreys C. J.; Sutton A. P. Electron-energy-loss spectra and the structural stability of nickel oxide: An LSDA+U study. Phys. Rev. B 1998, 57, 1505 10.1103/PhysRevB.57.1505.

