# Supplementary material for: Depth-Resolved Profile of the Interfacial Ferromagnetism in CaMnO3/CaRuO3 Superlattices
Source: Nano Lett. 2024 Nov 20;24(48):15195–201. doi: 10.1021/acs.nanolett.4c02087 (PMC11622370; doi:10.1021/acs.nanolett.4c02087)
Supplement: Supplementary file 1 — nl4c02087_si_001.pdf [file nl4c02087_si_001.pdf]

# *Supporting Information*

# Depth-resolved profile of the interfacial ferromagnetism in $CaMnO_3/CaRuO_3$ superlattices

Jay R. Paudel[1], Aria Mansouri Tehrani[2], Michael Terilli[3], Mikhail Kareev[3], Joseph Grassi[1], Raj K. Sah[1], Liang Wu[3], Vladimir N. Strocov[4], Christoph Klewe[5], Padraic Shafer[5], Jak Chakhalian[3], Nicola A. Spaldin[2], and Alexander X. Gray[1]

[1] *Physics Department, Temple University, Philadelphia, Pennsylvania 19122, USA*

[2] *Materials Theory, ETH Zurich, Wolfgang-Pauli-Strasse 27, CH-8093 Zürich, Switzerland*

[3] *Department of Physics and Astronomy, Rutgers University, Piscataway, New Jersey 08854, USA*

[4] *Swiss Light Source, Paul Scherrer Institute, 5232 Villigen, Switzerland*

[5] *Advanced Light Source, Lawrence Berkeley National Laboratory, Berkeley, California 94720, USA*

**email: axgray@temple.edu*

## Figure S1: Laboratory-based X-ray Diffraction (XRD) and Reflectivity (XRR)

Figure S1a (next page) shows the laboratory-based X-ray diffraction θ-2θ spectrum measured on the [4 u.c. $CaMnO_3$ / 4 u.c. $CaRuO_3$] ×10 superlattice. The (002) $LaAlO_3$ substrate peak appears at 2θ = 48°, consistent with prior studies [1,2]. The $0^{th}$-order superlattice peak ($SL_0$) is obscured by the substrate peak. The two $1^{st}$-order superlattice peaks ($SL_{-1}$) are observed at the symmetric angular positions of 44.65° and 51.40°, approximately ±3.4° from the $0^{th}$-order peak. The superlattice peaks exhibit shapes characteristic of high-quality single-crystalline superlattices. The spectrum shows the expected number (P − 2 = 8) of SL thickness fringes for P = 10 superlattice periods. Observation of the pronounced SL thickness fringes indicates that the superlattice layers are reasonably smooth.

Figure S1b shows the complementary laboratory-based angle-resolved XRR spectrum measured on the same superlattice. The data, characterized by distinct thickness fringes, indicate a high-quality interface. The best fit of the experimental spectrum, obtained using X-ray optical code, yields a total thickness for the superlattice of approximately 28.05 nm, which is quantitatively consistent with the synchrotron-based XRR results presented in the main text.

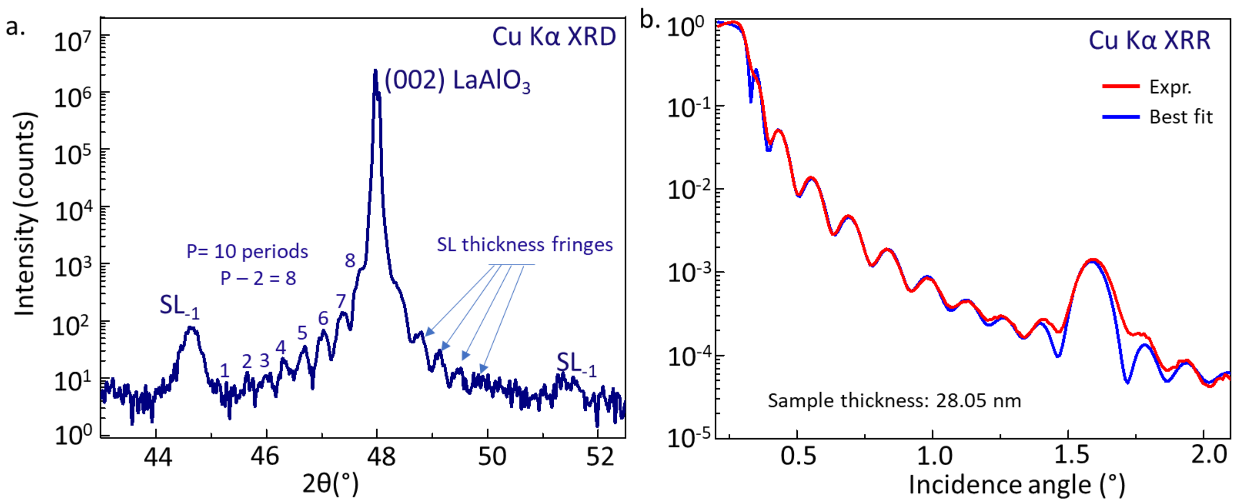


**Figure S1 a.** The X-ray diffraction (XRD) θ-2θ spectrum for the [4 u.c. $CaMnO_3$ / 4 u.c. $CaRuO_3$] ×10 superlattice. In this configuration, the 1$^{st}$-order superlattice peaks ($SL_{-1}$) are prominently visible on either side of the substrate peak, appearing at 44.65° and 51.40°, respectively. The superlattice exhibits the anticipated number of SL thickness fringes, amounting to $P - 2 = 8$ for $P = 10$ superlattice periods. **b.** The X-ray reflectivity (XRR) measurement conducted on the same superlattice. The experimental XRR data, characterized by distinct thickness fringes, indicates a high-quality interface. The fitting of this data using the theoretical model yields a total thickness for the superlattice of approximately 28.05 nm.

**Figure S2: Synchrotron-based Standing-Wave X-ray Photoelectron Spectroscopy (SW-XPS)**

Figure S2 shows the results of the SW-XPS measurements carried out at the soft-X-ray ARPES endstation [3] of the high-resolution ADRESS beamline at the Swiss Light Source [4]. In the SW-XPS technique, shown schematically in Figure S2a, Ångstrom-level depth resolution is facilitated by generating an X-ray standing-wave (SW) interference field within a periodic superlattice sample [5,6]. Once the X-ray SW field is established within the sample, it can be translated vertically (perpendicular to the sample's surface) by approximately half of the superlattice period by scanning (rocking) the grazing X-ray incidence angle across the Bragg condition. Photoemission intensities of various core-level photoemission peaks are then recorded

as a function of the X-ray incidence angle (rocking curves), thus facilitating element-specific depth profiling of the sample.

Figures S2b-d show experimental rocking-curve spectra for the integrated intensities of the Ca 2*p*, O 1*s*, and Ru 3*d* core-level peaks (red curves in the upper panels) and the best fits to the experimental data, calculated using an X-ray optical code [7,8] (blue curves in the lower panels). The extracted depth profile of the superlattice, yielding the values of the individual layer thicknesses and interface roughness (interdiffusion), is shown in panel e. The results are in quantitative agreement with both the lab-based XRR measurements shown in Supporting Figure S1 and the synchrotron-based resonant and non-resonant XRR measurements presented in Figure 1 of the main text.

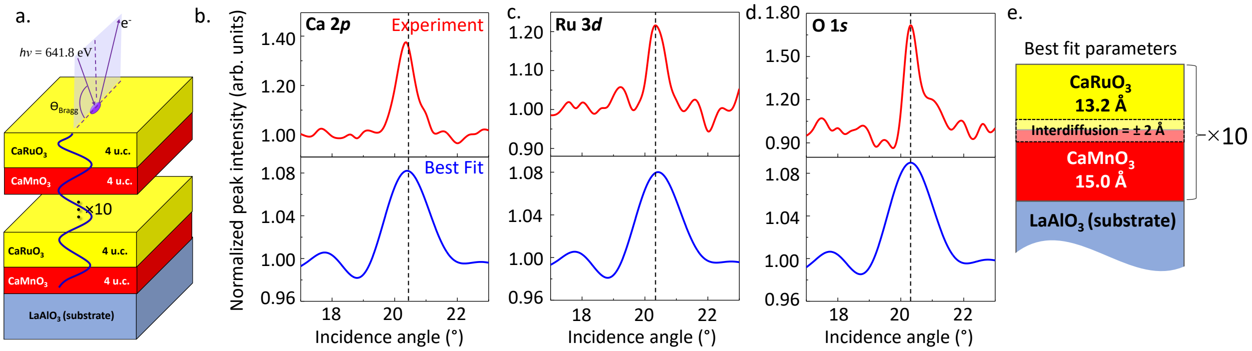


**Figure S2 a.** Schematic diagram of the standing-wave X-ray photoelectron spectroscopy (SW-XPS) experiment and the investigated superlattice structures consisting of 10 $CaMnO_3$/$CaRuO_3$ bilayers grown epitaxially on a $LaAlO_3$(001) substrate, with each bilayer consisting of 4 u.c. of $CaMnO_3$ and 4 u.c. of $CaRuO_3$. **b-d.** Best fits between the experimental (red curve, top panel) and calculated (blue curve, bottom panel) SW rocking curves for Ca 2*p*, O 1*s*, and Ru 3*d* core levels, respectively, for the superlattice. **e.** The resultant depth profile yielding the values of the individual layer thicknesses and interface roughness (interdiffusion).

**Figures S3 and S4: Hard X-ray Photoelectron Spectroscopy (HAXPES) Characterization**

The nominal chemical composition of the superlattices was confirmed using bulk-sensitive HAXPES measurements carried out using a laboratory-based spectrometer equipped with a 5.41 keV monochromated X-ray source and a Scienta Omicron EW4000 high-energy hemispherical analyzer. Figure S3 below shows wide-energy range HAXPES survey spectra for the $CaRuO_3$-terminated (red line) and $CaMnO_3$-terminated (blue line) superlattices. The presence of all expected elements (Ca, Mn, O, Ru, and C from the surface-adsorbed contaminant C/O layer) is confirmed by the presence of corresponding core-level peaks. Measurements were carried out at room temperature.

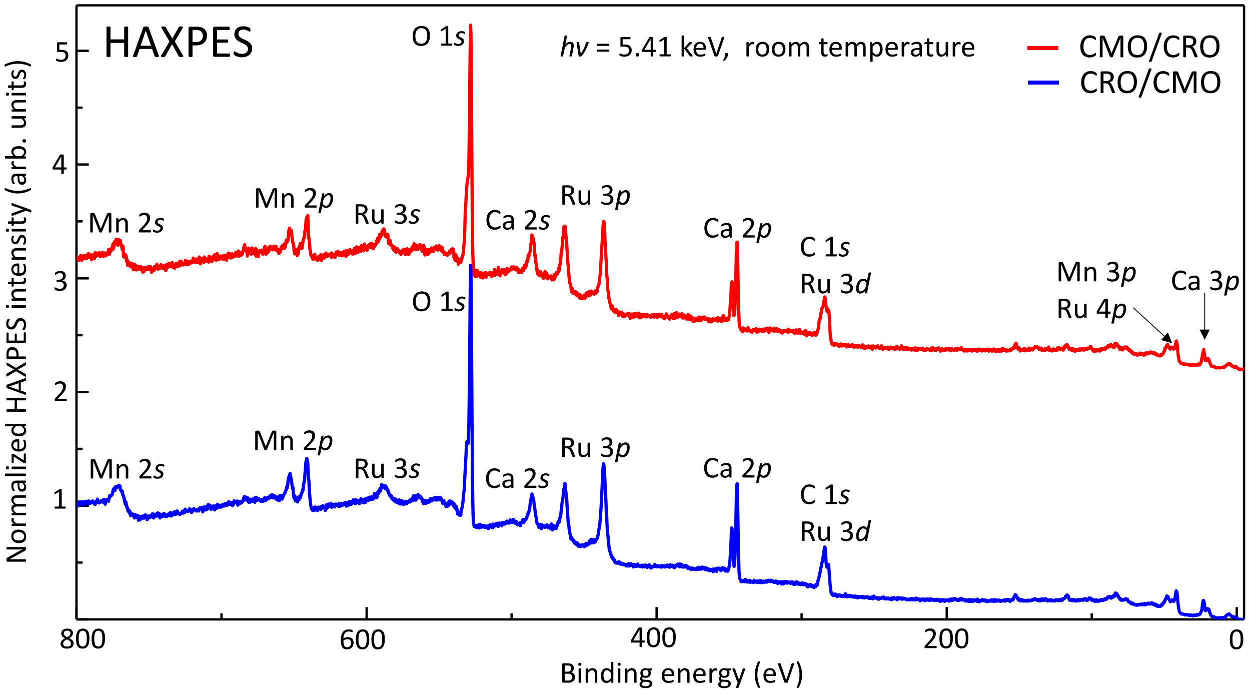


**Figure S3.** Wide-energy-range HAXPES survey spectra for the $CaRuO_3$ terminated (CMO/CRO) (red line) and $CaMnO_3$ terminated (CRO/CMO) (blue line) superlattices.

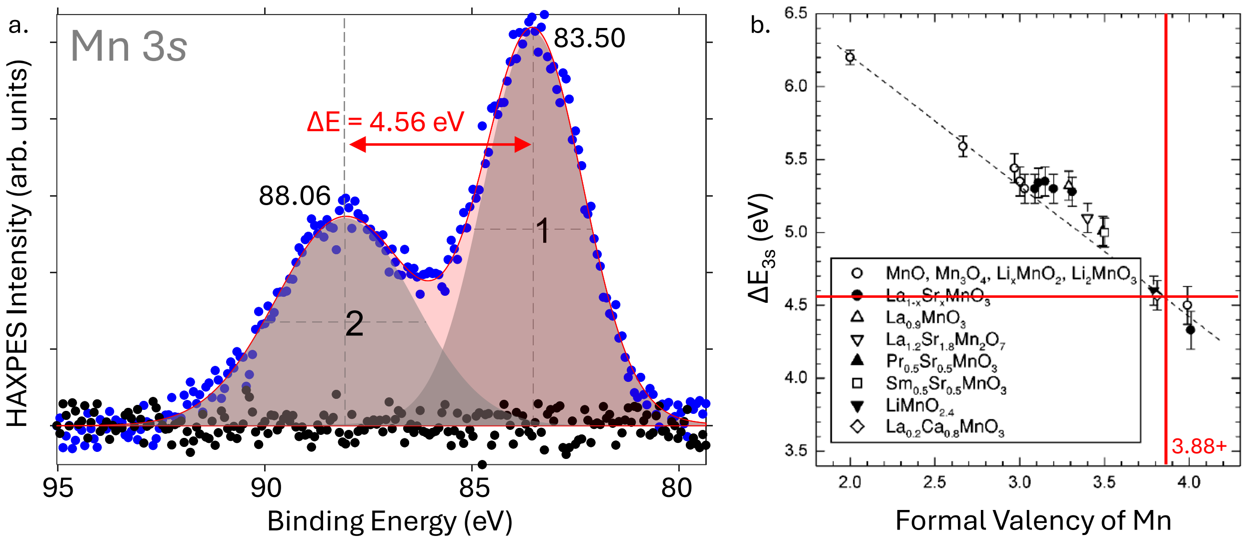


**Figure S4. a.** Mn 3*s* core-level spectrum measured with the photon energy of 5.4 keV. Magnitude of the splitting (4.56 eV), quantified using peak fitting, can be related to the effective valence state of the Mn ions in the film (see panel **b.** from Ref.10). The measured value of splitting corresponds to the formal Mn valency of approximately +3.88, yielding a nearly-stoichiometric composition of $CaMnO_{2.94}$ (δ = 0.06).

To quantify the Mn valence state in $CaMnO_3$ and the film's stoichiometry, we performed Mn 3*s* HAXPES spectroscopy measurements on the $CaMnO_3$-terminated superlattice (CRO/CMO). In 3*d* transition metals, 3*s* core-level splitting occurs due to exchange coupling between the 3*s* core hole and 3*d* electrons. As the Mn ion's valence state decreases, the energy splitting between the two Mn 3*s* multiplet components increases [9]. Consequently, 3*s* splitting is highly sensitive to changes in the Mn valence state and has been widely used to determine it [10].

The results of our measurements and data fitting are presented in Figure S4 above. The extracted Mn 3*s* multiplet splitting value is 4.56 eV, corresponding to an effective Mn valence of approximately +3.88. This indicates a near-stoichiometric composition of $CaMnO_{2.94}$ (δ = 0.06), where δ represents the deviation from stoichiometry. Accounting for the reduction of Mn valence due to charge transfer from Ru brings δ even closer to zero. These results demonstrate the high quality of the sample, with only a minimal presence of oxygen vacancies in $CaMnO_3$.

**Figure S5: X-ray Absorption Spectroscopy (XAS) in the Total Electron Yield (TEY) Mode**

In order to directly probe the magnetic moment at the two types of interfaces ($CaMnO_3/CaRuO_3$ and $CaRuO_3/CaMnO_3$) in the superlattice, we conducted a comparative study using XAS/XMCD in the total electron yield (TEY) detection mode. TEY is a surface-sensitive XAS modality with an average probing depth of 2-5 nm, which decays exponentially from the surface into the bulk due to the short electron mean free path in solids [11,12].

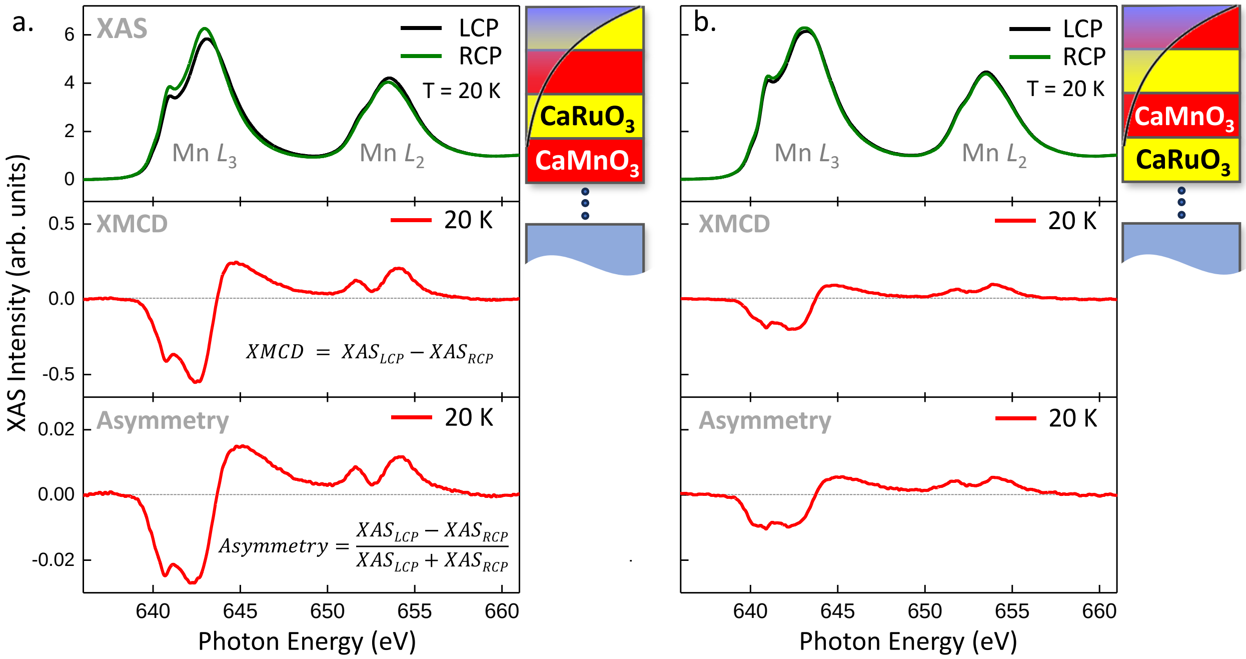


**Figure S5 a.** Circular polarization-dependent Mn $L_{2,3}$ X-ray absorption spectroscopy (XAS) spectra measured at T = 20 K (~80 K below $T_c$) on a $[CaMnO_3/CaRuO_3]\times10$ superlattice. XMCD and asymmetry plots show strong magnetic signal. **b.** Similar measurements carried out on a $[CaRuO_3/CaMnO_3]\times10$ superlattice reveal significant suppression of the magnetic signal (by a factor of ~2.7). Schematics of the samples' topmost layers with overlaid plots of the TEY signal intensities, which decrease exponentially with depth, illustrate sensitivity to the two types of interfaces. The probing depth is assumed (conservatively) to be 5 nm.

An additional superlattice sample was grown in the same batch as the original superlattice, terminated with a $CaMnO_3$ layer instead of $CaRuO_3$. The XAS/XMCD measurements of the original $CaMnO_3/CaRuO_3$ sample (terminated with $CaRuO_3$) shown in panel **a.** are most sensitive to the $CaMnO_3/CaRuO_3$ ('top' type) interface. Conversely, a similar measurement of the $CaRuO_3/CaMnO_3$ sample (terminated with $CaMnO_3$) shown in panel **b.** is most sensitive to the $CaRuO_3/CaMnO_3$ ('bottom' type) interface. The measurements reveal a significantly weaker (×**2.7**) magnetic signal for the $CaRuO_3/CaMnO_3$ ('bottom' type) interface, qualitatively consistent with our $q_z$-dependent XRR measurements shown in Figure 2b of the main text.

This experimentally determined **2.7**-fold reduction of the XMCD signal can also be quantitatively predicted by calculating the ratio of the XMCD signals for the two differently terminated superlattices at a given probing depth.

For an accurate XMCD signal ratio calculation, it is essential to consider consistent probing depths for both samples and account for the appropriate number of interfaces within those depths. As an illustration, the schematic diagram below shows the two samples with different terminations and labels the interfaces that must be considered in the model for a probing depth of $\tau$ = 5 nm: interfaces **a**, **b**, and **c** for the $CaRuO_3$-terminated sample and **a'**, **b'**, and **c'** for the $CaMnO_3$-terminated sample.

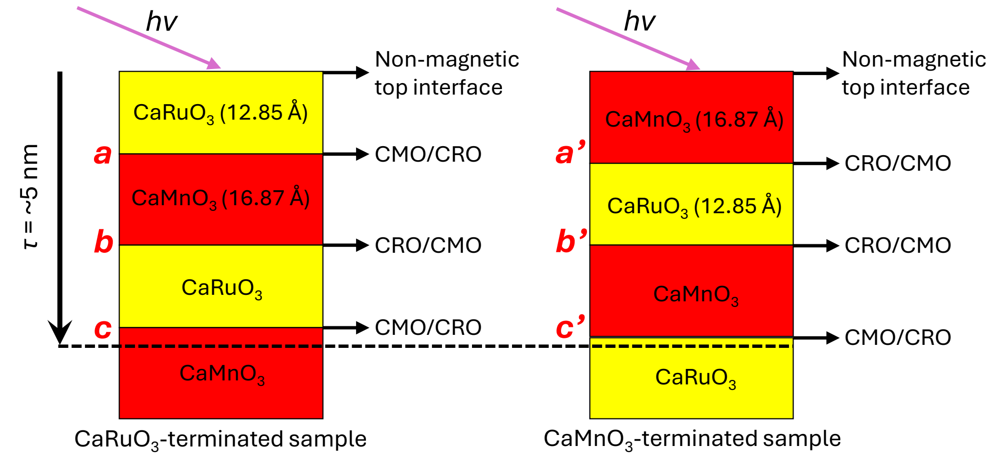

Applying this model to the calculation of the XMCD signal ratio between the two samples, assuming the interfaces are magnetically **asymmetric** and only the $CaMnO_3/CaRuO_3$ interface is magnetic, gives:

$$XMCD\ Ratio = \frac{1 + 0 + \frac{1}{e^{(16.87\ \text{Å}+12.85\text{Å}/\tau)}}}{0 + \frac{1}{e^{(16.87\ \text{Å}+12.85\text{Å})/\tau)}} + 0} = 2.8$$

For an escape depth of $\tau$ = 5 nm and under the assumption of magnetically asymmetric interfaces, this yields an XMCD ratio of **2.8**, which is within 4% of our experimental value of **2.7**.

Conversely, a similar calculation for the case where both the "top" and "bottom" $CaMnO_3$ interfaces are magnetically **symmetric** gives:

$$XMCD\ Ratio = \frac{1 + \frac{1}{e^{(3*3.73\ \text{Å}/\tau)}} + \frac{1}{e^{(16.87\ \text{Å}+12.85\text{Å}/\tau)}}}{\frac{1}{e^{(3*3.73\ \text{Å}/\tau)}} + \frac{1}{e^{(16.87\ \text{Å}+12.85\text{Å})/\tau)}} + \frac{1}{e^{(16.87\ \text{Å}+12.85\text{Å}+3*3.73\ \text{Å})/\tau)}}} = 1.3$$

For an escape depth of $\tau$ = 5 nm and assuming magnetically symmetric interfaces, this yields an XMCD ratio of 1.3. This value is more than a factor of two smaller than the XMCD ratio we observe for both normalized and non-normalized XMCD signals (**2.7**).

**Figure S6: XAS on the Bulk-like Reference $CaMnO_3$ Film in the Luminescence Yield Mode**

The thickness of the individual $CaMnO_3$ layers in the superlattices plays a crucial role in determining the observed effective valence state of Mn. In previous studies, which mostly used thicker $CaMnO_3$ layers - typically 10 to 15 unit cells - the ratio of interfacial $CaMnO_3$ layers to bulk-like $CaMnO_3$ layers was relatively small, so the Mn valence state was predominantly determined by the bulk properties. In contrast, our superlattices consist of significantly thinner

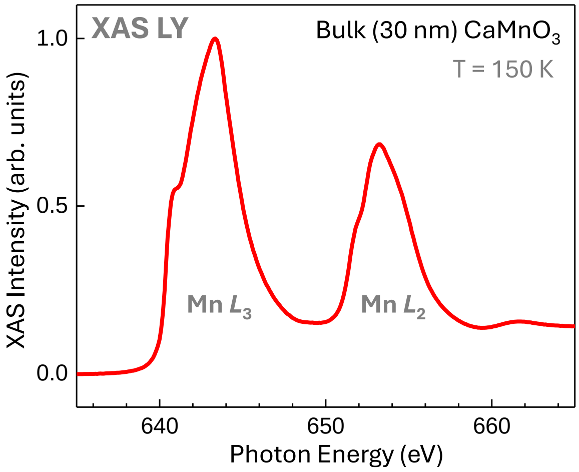


**Figure S6.** Mn $L_{2,3}$ XAS spectrum measured on a bulk-like 30 nm-thick $CaMnO_3$ film grown on an $LaAlO_3$ substrate. Measurements were carried out using the bulk-sensitive luminescence yield (LY) detection mode at T = 150 K (the LY mode does not work at very low temperatures). Right-circular and left-circular polarized spectra were averaged to obtain the resultant spectrum.

$CaMnO_3$ layers (~4 unit cells). This results in a much higher ratio of interfacial $CaMnO_3$ to bulk $CaMnO_3$ compared to previous studies. Consequently, the reduced Mn state at the interfaces, either due to charge transfer effects or the presence of interfacial oxygen vacancies as suggested by our theoretical calculations, has a more significant impact on the observed effective valence state of Mn throughout the superlattice.

To demonstrate this experimentally, we have characterized a 30 nm-thick (bulk-like) $CaMnO_3$ film grown using the same parameters and conditions as those used for the $CaMnO_3$ in our superlattices. The XAS spectrum measured in bulk-sensitive luminescence yield (LY) detection mode, which is sensitive to the entire thickness of the film, is shown in Figure S6 above. The spectrum exhibits a lineshape characteristic of a predominantly $Mn^{4+}$ valence state, with only a minor $Mn^{3+}$-like component on the lower-photon-energy side. A direct comparison with the superlattice spectra shown in Figure S5 suggests that the reduced Mn state observed in the superlattice samples is likely due to interfacial effects rather than intrinsic oxygen deficiency from the growth process.

**Figure S7: Comparing Interface- and Bulk-Sensitive XAS Spectra of the Superlattices**

In order to ascertain the origin of the differences between the Mn $L_{2,3}$ XAS TEY spectra of the [$CaMnO_3$/$CaRuO_3$]×10 and [$CaRuO_3$/$CaMnO_3$]×10 superlattices shown in Figure S5, we carried out additional bulk-sensitive XAS measurements on both samples using the LY detection mode, which probes the entire depth of the superlattice. Figure S7a (top panel) shows the Mn $L_{2,3}$ spectra measured in the TEY mode, which differentiates between the two types of interfaces due to the limited probing depth. Here, we observe the difference between the 4+ and 3+ concentrations for the two samples. The bottom panel (S7b) shows the XAS LY spectra that reflect the average valence state of Mn in the entire superlattice, and they overlap perfectly.

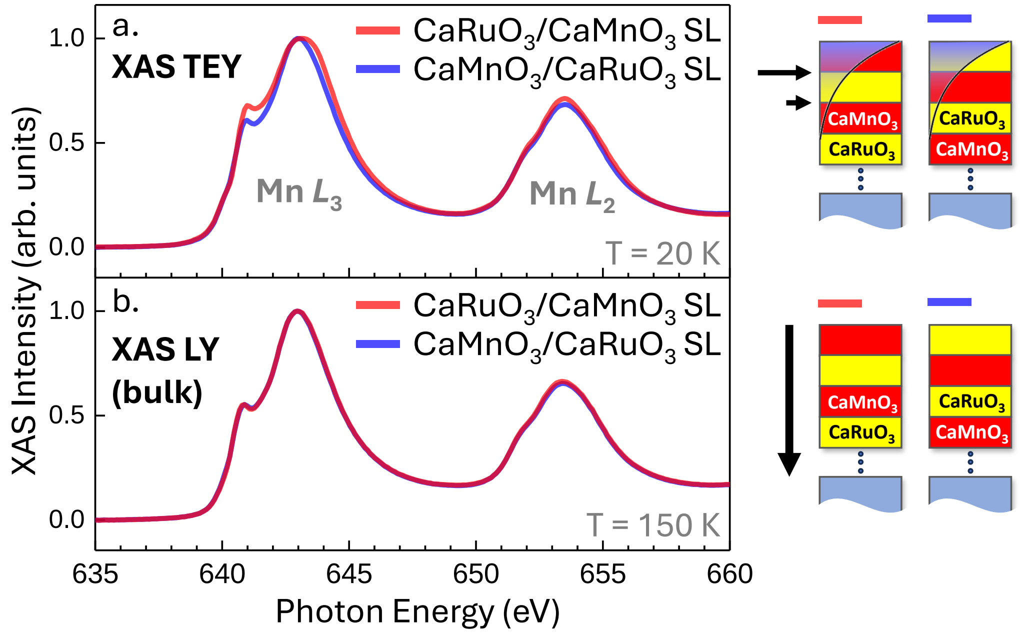


**Figure S7. a.** Circular polarization-averaged Mn $L_{2,3}$ X-ray absorption spectroscopy (XAS TEY) spectra measured at T = 20 K on the [$CaRuO_3$/$CaMnO_3$]×10 superlattice (red solid line) and the [$CaMnO_3$/$CaRuO_3$]×10 superlattice (blue solid line). The TEY mode differentiates between the two types of interfaces due to its limited probing depth (see schematics on the right side). **b.** Same spectra measured using bulk-sensitive LY detection mode (at T = 150 K), which probes the entire depth of the superlattices, and thus reflect the average valence state of Mn in the entire superlattice.

This strongly suggests that the difference in the Mn 4+ and 3+ concentrations between the two superlattices (observed in the top panel) originates at the interfaces. Furthermore, since the average effective valence states in both superlattices are the same, it is reasonable to deduce that the asymmetry between the "top-like" and "bottom-like" interfaces propagates consistently throughout the superlattice.

**Figure S8: Symmetric magnetic interfaces – best fits for the constrained models**

To verify the observed difference (asymmetry) in the magnitudes of the Mn magnetic moments between the top and bottom $CaMnO_3$ interfaces, we repeated the fitting of the $q_z$-dependent XMCD asymmetry spectra at the Mn $L_3$ and $L_2$ edges with an additional constraint, requiring the magnitudes of $\Delta\beta_m$ to be the same for both interfaces while varying together in the model. The results of these calculations are shown in Figures S8a (Mn $L_3$ edge) and S7b (Mn $L_2$ edge) on the next page. While the quality of these fits was not significantly worse compared to the best fits in Figure 2 of the main text (notably, inconsistencies were observed in the intensities of peaks labeled A, B, C, D, and E, as well as shifts in peaks C and F), the Mn $L_3$ and $L_2$ XRR XMCD data yielded different, inconsistent, and non-physical values for the thickness of the magnetic layer (0.86–1.36 Å). This discrepancy indicates that the fits in Figures S8a and S8b are likely *local minima* rather than the global minimum, underscoring the reliability of our original results (Figure 2 in the main text), which predict consistent and physical magnetic layer thicknesses from both Mn $L_2$ and $L_3$ independent fittings, as well as between the top and bottom interfaces.

As an additional check, we repeated the calculations with the thickness of the two symmetric magnetic interfaces fixed in the model to the best-fit value obtained in the original calculation shown in Figure 2 of the main text. However, this approach produced an even worse fit for the Mn $L_3$ edge data and did not converge at all for the Mn $L_2$ edge fit, as depicted in Figures S8c and S8d

below. This suggests that, in this particular case, the model became overconstrained, with too few degrees of freedom to achieve the optimal fit. Furthermore, the condition for the interfacial magnetic symmetry has put the model too far away from the global minimum, especially for the Mn $L_2$ edge data (panel S8d).

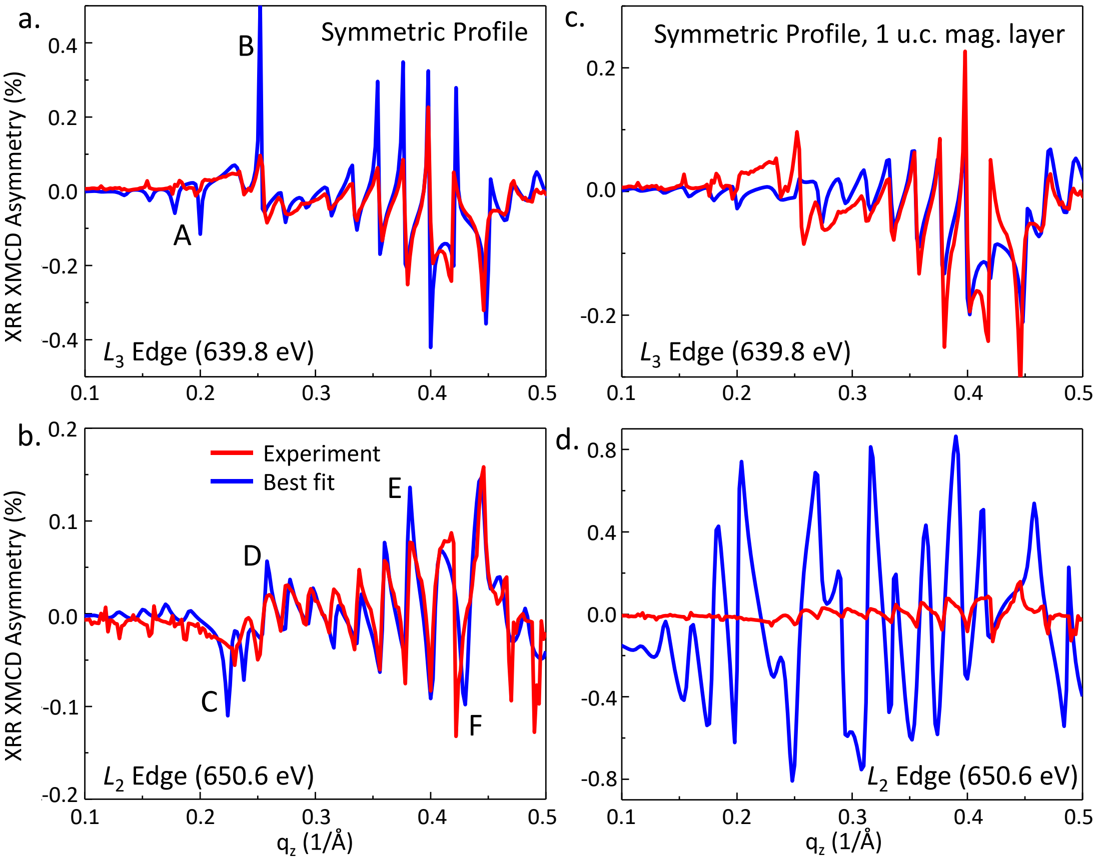


**Figure S8. a-b.** $q_z$-dependent XMCD asymmetry spectra (red curves) and the best-fit results to the experimental data (blue curves) for the Mn $L_3$ (panel a) and $L_2$ (panel b) absorption edges. The fitting was carried out with a constraint that required the magnitudes of $\Delta\beta_m$ to be the same for both interfaces, while being optimized together during the fitting process, along with variation in magnetic layer thickness. **c-d.** Additional fits, repeated with the thickness of the two symmetric magnetic interfaces constrained (fixed) in the model to the best-fit value obtained in the original calculation shown in Figure 2 of the main text.

**Figure S9: Possible deviations from the nominal interface structure and the resultant Mn magnetic moments**

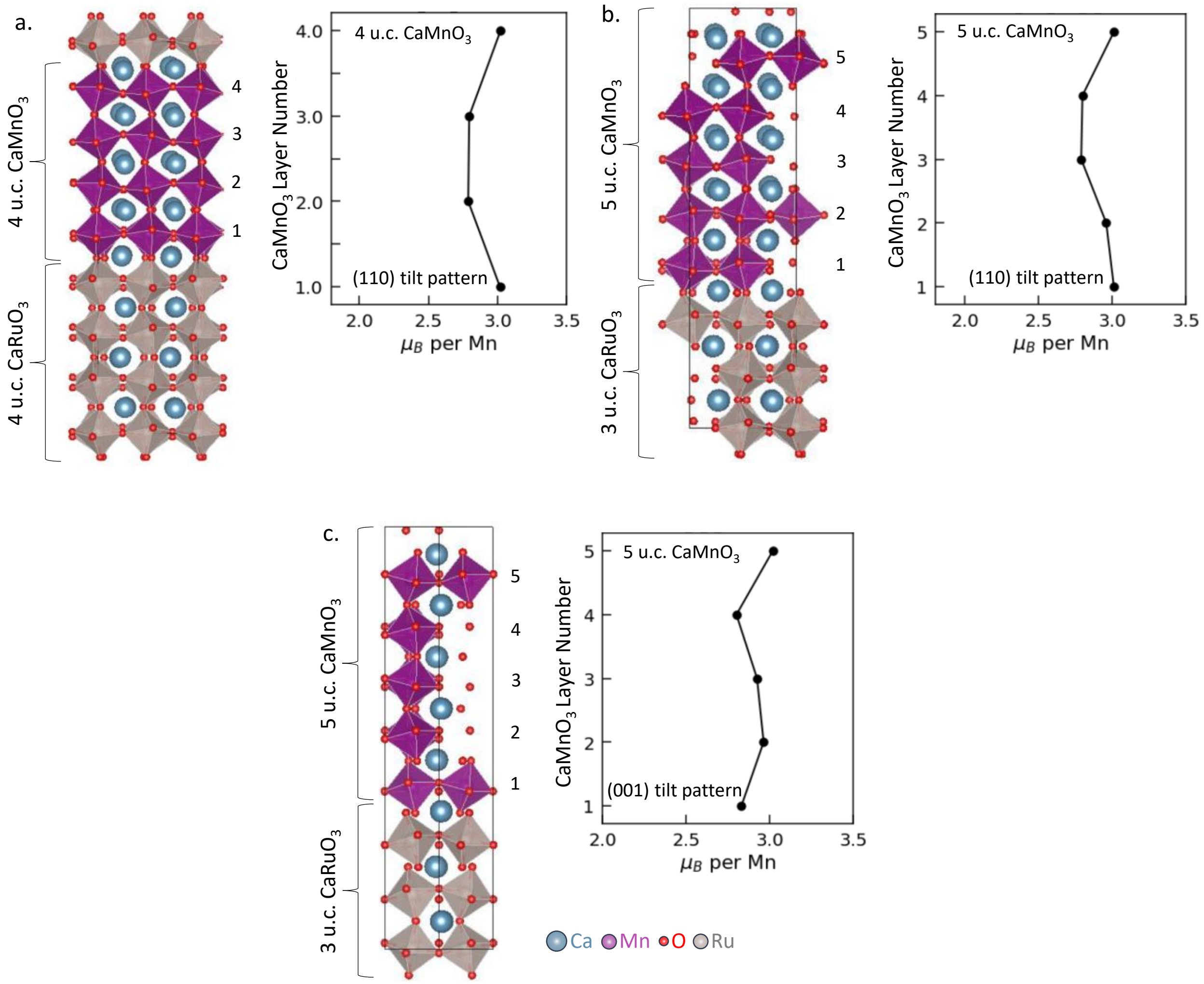


**Figure S9. a.** Crystal structure of the 4 u.c. / 4 u.c. superlattice with [110] oxygen octahedral tilt patterns in the interfacial $CaMnO_3$ layer and the resultant layer-resolved Mn magnetic moments. **b.** Similar calculations for the 3 u.c. / 5 u.c. superlattice. c. Similar calculations for the 3 u.c. / 5 u.c. superlattice with nominal [001] oxygen octahedral tilt patterns in the interfacial $CaMnO_3$ layer.

To investigate or rule-out other possible origins of the observed magnetic asymmetry between the top and bottom $CaMnO_3$ interfacial layers, we repeated our calculation procedure for supercells with several plausible deviations from the nominal structure shown previously in Figure

3a. Thus, in Figure S9a on the previous page, we consider the nominal 4 u.c./4 u.c. superlattice structure, but with [110] oxygen octahedral tilt patterns in the interfacial $CaMnO_3$ layer. The right panel shows the resultant calculated layer-resolved Mn magnetic moments in the $CaMnO_3$ layer. Interestingly, we observed no significant magnetic asymmetry between the top and bottom interfacial $CaMnO_3$ layers.

We then repeated our calculation for a similar structure, but with an odd number of unit cells (5 u.c. and 3 u.c., respectively) of $CaMnO_3$ and $CaRuO_3$. The proposed structure and the corresponding Mn magnetic moment calculations are shown in Figure S9b. An additional calculation for this structure, but with [001] oxygen octahedral tilt patterns in the interfacial $CaMnO_3$ layer, are shown in Figure S9c. In each case, our calculations showed no significant magnetic asymmetry between the top and bottom interfacial $CaMnO_3$ layers.

**Details of the DFT Calculations**

We used the VASP DFT implementation [13] with the LDA exchange correlation functional, combined with a Hubbard U of 3 eV applied to the Mn 3*d* orbitals using the Dudarev approach [14], and the default VASP pseudopotentials with the following electrons included in the valence manifold: Ca $3s^2$, $3p^6$, $4s^2$; Mn $3p^6$, $4s^2$, $3d^5$; O $2s^2$, $2p^4$; and Ru $4p^6$, $4d^7$, $5s^2$. Our superlattices were constructed of four layers of $CaMnO_3$ alternating with four layers of $CaRuO_3$ (see Figure 3a in the main text), corresponding to both [001] and [110] growth directions, as well as mixed heterostructures with alternating [001]- and [110]-oriented slabs. We used an in-plane periodically repeated unit cell of size $\sqrt{2} \times \sqrt{2}$ times the simple cubic perovskite cell to accommodate rotations and tilting of the oxygen octahedra, with the in-plane lattice parameters set to the experimental lattice parameter of $LaAlO_3$. The $c$ lattice parameter and the internal coordinates were adjusted to their lowest energy values using an energy cutoff of 800 eV, a $k$-point mesh of $12\times12\times1$ for the

smallest (80 atom) supercell adjusted for larger supercells and convergence criteria of $1\times10^{-7}$ eV and $1\times10^{-3}$ eV/A for the electronic and ionic relaxations, respectively. For the internal coordinate relaxations, numerous starting structures with different likely octahedral tilt patterns were used to ensure a thorough sampling of the structural landscape, and in all cases a range of likely magnetic orderings was explored.

## REFERENCES


1. Flint, C. L.; Jang, H.; Lee, J.-S.; N'Diaye, A. T.; Shafer, P.; Arenholz, E.; Suzuki, Y. Role of polar compensation in interfacial ferromagnetism of $LaNiO_3/CaMnO_3$ superlattices. *Phys. Rev. Mater.* **2017**, 1, 024404.
2. Chandrasena, R. U.; Flint, C. L.; Yang, W.; Arab, A.; Nemšák, S.; Gehlmann, M.; Özdöl, V. B.; Bisti, F.; Wijesekara, K. D.; Meyer-Ilse, J.; Gullikson, E.; Arenholz, E.; Ciston, J.; Schneider, C. M.; Strocov, V. N.; Suzuki, Y.; Gray, A. X. Depth-resolved charge reconstruction at the $LaNiO_3/CaMnO_3$ interface. *Phys. Rev. B* **2018**, 98, 155103.
3. Strocov, V. N.; Wang, X.; Shi, M.; Kobayashi, M.; Krempasky, J.; Hess, C.; Schmitt, T.; Patthey, L. Soft-X-ray ARPES facility at the ADRESS beamline of the SLS: concepts, technical realization and scientific applications. *J. Synchrotron Rad.* **2014**, 21, 32-44.
4. Strocov, V. N.; Schmitt, T.; Flechsig, U.; Schmidt, T.; Imhof, A.; Chen, Q.; Raabe, J.; Betemps, R.; Zimoch, D.; Krempasky, J.; Wang, X.; Grioni, M.; Piazzalunga A.; Patthey, L. High-resolution soft X-ray beamline ADRESS at the Swiss Light Source for resonant inelastic X-ray scattering and angle-resolved photoelectron spectroscopies. *J. Synchrotron Rad.* **2010**, 17, 631-643.
5. Yang, S.-H.; Mun, B. S.; Kay, A. W.; Kim, S.-K.; Kortright, J. B.; Underwood, J. H.; Hussain, Z.; Fadley, C. S.; Depth-Resolved Photoemission Spectroscopy from Surfaces and Buried Layers with Soft X-Ray Standing Waves, *Surf. Sci. Lett.* **2000**, 461, L557.
6. Kuo, C.-T.; Conti, G.; Rault, J. E.; Schneider, C. M.; Nemšàk, S.; Gray, A. X. Emergent phenomena at oxide interfaces studied with standing-wave photoelectron spectroscopy. *J. Vac. Sci. Technol. A* **2022**, 40, 020801.

7. Yang, S.-H.; Gray, A. X.; Kaiser, A. M.; Mun, B. S.; Sell, B. C.; Kortright, J. B.; Fadley, C. S. Making use of X-ray optical effects in photoelectron-, Auger electron-, and X-ray emission spectroscopies: Total reflection, standing-wave excitation, and resonant effects. *J. Appl. Phys.* **2013**, 113, 073513.

8. Karslıoğlu, O.; Gehlmann, M.; Müller, J.; Nemšák, S.; Sethian, J. A.; Kaduwela, A.; Bluhm, H.; Fadley, C. S. An Efficient Algorithm for Automatic Structure Optimization in X-ray Standing-Wave Experiments. *J. Electron Spectrosc. Rel. Phenom.* **2019**, 230, 10-20.

9. Fadley, C. S.; Shirley, D. A. Multiplet Splitting of Metal-Atom Electron Binding Energies. *Phys. Rev. A* **1970**, 2, 1109-1120.

10. Galakhov, V. R.; Demeter, M.; Bartkowski, S.; Neumann, M.; Ovechkina, N. A.; Kurmaev, E. Z.; Lobachevskaya, N. I.; Mukovskii, Y. M.; Mitchell, J.; Ederer, D. L. Mn 3*s* exchange splitting in mixed-valence manganites. *Phys. Rev. B* **2002**, 65, 113102.

11. Nakajima, R.; Stöhr, J.; Idzerda, Y. U.; Electron-yield saturation effects in *L*-edge X-ray magnetic circular dichroism spectra of Fe, Co, and Ni. *Phys. Rev. B* **1999**, *59*, 6421.

12. Jablonski, A.; Powell, C. J. Practical expressions for the mean escape depth, the information depth, and the effective attenuation length in Auger-electron spectroscopy and X-ray photoelectron spectroscopy. *J. Vac. Sci. Technol. A* **2009**, 27, 253.

13. Kresse, G.; Furthmüller, J. Efficient iterative schemes for *ab initio* total-energy calculations using a plane-wave basis set. *Phys. Rev. B* **1996**, *54*, 11169.

14. Dudarev, S. L.; Botton, G. A.; Savrasov, S. Y.; Humphreys, C. J.; Sutton, A. P. Electron-energy-loss spectra and the structural stability of nickel oxide: An LSDA+U study, *Phys. Rev. B* **1998**, *57*, 1505.
